# Rethinking parvalbumin: From passive marker to active modulator of hippocampal circuits

**DOI:** 10.1016/j.ibneur.2025.10.005

**Published:** 2025-10-19

**Authors:** Na Zhang, Bo-Wu Hu, Xiao-Ming Li, Huiqian Huang

**Affiliations:** a Department of Neurobiology and Department of Psychiatry, the Second Affiliated Hospital, Zhejiang University School of Medicine, Hangzhou 310058, China; b NHC and CAMS Key Laboratory of Medical Neurobiology, MOE Frontier Science Center for Brain Science and Brain-machine Integration, School of Brain Science and Brain Medicine, Zhejiang University, Hangzhou 310058, China

**Keywords:** Parvalbumin, Hippocampal circuits, Passive marker, High-frequency firing, Neuroprotection, Diseases

## Abstract

Parvalbumin (PV)-expressing interneurons are critical regulators of neural circuit dynamics, and for decades, the PV protein has served as their definitive molecular marker. This review confronts a central, yet underappreciated, paradox: the incongruity of a kinetically slow Ca²⁺ buffer (PV) being the defining feature of the brain's fastest-spiking neurons. We synthesize evidence from molecular biophysics, genetics, in vivo circuit analysis, and disease modeling to dissect the dual role of PV as both a cellular marker and an active functional regulator. We argue that PV’s slow kinetics are not a coincidence but a crucial adaptation that shapes short-term synaptic plasticity, protects against metabolic stress during high-frequency firing, and allows the circuit to shift between states of plasticity and stability. This reframing resolves the paradox by demonstrating how a "slow" molecule is essential for "fast" neuronal function. Furthermore, we highlight that dysfunction of the PV system is a convergent hub of pathology in numerous neurological and psychiatric disorders, including schizophrenia, epilepsy, and Alzheimer's disease. By moving beyond its identity as a passive marker, we establish PV as an active modulator of neural computation and a potential therapeutic target for restoring network function in disease.

## Introduction

### The parvalbumin paradox: a core question

Within the intricate architecture of the brain's neural circuits, parvalbumin-expressing (PV^+^) interneurons constitute a cornerstone of inhibitory control (Hijazi et al., 2023). These fast-spiking (FS) GABAergic neurons are indispensable for shaping network dynamics, generating high-frequency oscillations, and maintaining the delicate balance between excitation and inhibition (Benamer et al., 2020, Hu et al., 2014, Okur et al., 2024, Sohal et al., 2009). Their defining molecular feature is the expression of the protein parvalbumin (PV), a highly specific and reliable marker for this cell type. First identified in the 1980s (e.g. Celio, 1986), PV has been since established as a defining feature of a major interneuron class (Celio, 1986). However, the functional significance of PV– beyond its role of simply labeling cell identity – remains an intriguing paradox in neurobiology.

PV is a calcium-binding protein (CaBPs) act as slow calcium buffer (Caillard et al., 2000), yet it is prominently expressed in neurons specialized in fast spiking and synaptic output (Eggermann and Jonas, 2011). This kinetic mismatch is central to the paradox: a fast-firing neuron generates rapid, transient spikes in intracellular Ca²⁺, but PV is too slow to buffer the initial, sharp influx of ions that triggers neurotransmission. Instead, its primary role is to bind Ca²⁺ after the peak, thereby shaping the decay of the transient and managing residual calcium during repetitive firing (Schwaller, 2007). This observation presents a central puzzle: why would a neuron specialized for rapid signaling express a kinetically slow protein? This raises a fundamental question: Is the association between fast-firing neurons and a slow-acting protein an evolutionary coincidence, or does it reflect a co-evolved adaptive mechanism where PV's unique properties are essential for the neuron's specialized function? In other words, is parvalbumin merely a passive marker of the PV^+^ neuron's identity, or is it an active component of the machinery that enables and sustains its fast-spiking capabilities? Resolving whether PV shapes neuronal physiology or simply correlates with it remains critical for understanding interneuron function.

### The hippocampus: an ideal arena for dissecting the paradox

The hippocampus provides a uniquely powerful model system for dissecting the relationship between PV protein function and PV^+^ neuron function. Its well-defined microcircuitry, comprising the dentate gyrus (DG), CA3, and CA1 subfields, allows for the precise study of information flow and its modulation by local interneurons (Hu et al., 2014). Furthermore, the hippocampus harbors a rich diversity of PV^+^ neuron subtypes, each with distinct anatomical targets and functional roles. The primary subtypes include basket cells (BCs), chandelier cells (ChCs), also known as axo-axonic cells, oriens-lacunosum moleculare (OLM) cells, and bistratified cells (BiCs) (Bartholome et al., 2020). This subtype diversity offers a natural experimental framework: a robust hypothesis for PV's function must be applicable across these varied cellular and circuit contexts.

The functional significance of this system is profound. Hippocampus-dependent behaviors, including spatial navigation, episodic memory, and the regulation of emotion and anxiety, critically depend on the precise inhibitory control exerted by PV^+^ neurons (Cummings et al., 2021, Topolnik and Tamboli, 2022). These neurons are central to maintaining the excitatory/inhibitory (E/I) balance and orchestrating network oscillations (e.g., theta and gamma rhythms), which underlie these cognitive functions. The ventral hippocampus serves as a good example, where PV^+^ neurons are strongly implicated in the regulation of local inhibitor microcircuitry and thus emotional states like anxiety (Jimenez et al., 2018, Okur et al., 2024, Sohal et al., 2009, Tiwari et al., 2024, Volitaki et al., 2024). Consequently, dysfunction of the PV system is a convergent pathological feature in numerous hippocampal-related disorders, including schizophrenia, temporal lobe epilepsy, autism spectrum disorders, and early-stage Alzheimer's disease (Arime et al., 2024, Hernández-Frausto et al., 2023, Janickova et al., 2020, Yekhlef et al., 2015).

### Scope of this review

This review will critically evaluate the existing evidence to dissect the dual role of parvalbumin as both a cellular marker and a functional protein. The central objective is to move beyond correlation and probe the causal links between PV's molecular properties and the electrophysiological, circuit, and behavioral functions of the neurons that express it, with a specific focus on the diverse subtypes within the hippocampus. We will address the core question: is the PV protein merely a reliable label for a neuron that has developmentally committed to a fast-spiking fate, or do its unique calcium buffering kinetics actively shape and sustain that neuron's signature properties, plasticity, and vulnerability in disease? To achieve this, we first summarize PV’s molecular properties, then examine the anatomy and physiology of hippocampal PV⁺ interneurons. evaluate evidence that PV modulates neuronal dynamics, outline experimental strategies to probe its function, and conclude by proposing PV's dual role as both an identity marker and functional regulator. Additionally, we discuss clinical implications and highlight outstanding questions in the field.

## Parvalbumin: molecular properties and functional consequences

### Structural basis and biochemical kinetics

PV is a small (∼12 kDa), acidic protein of the EF-hand family, capable of binding both calcium (Ca^2+^) and magnesium (Mg^2+^) ions (Schwaller, 2010, Veshchitskii and Merkulyeva, 2023). It is characterized by three helix-loop-helix motifs, designated as the AB, CD, and EF domains (Schwaller, 2010, Strynadka and James, 1989). The CD and EF domains contain the canonical EF-hand loops that serve as the primary binding sites for divalent cations, accommodating both Ca^2+^ and magnesium (Mg^2+^) ions. A key structural feature is the allosteric role of the AB domain, which, despite not binding Ca²⁺ itself, is necessary for parvalbumin protein’s stability and maintaining high affinity for cation binding (Schwaller, 2010, Thépaut et al., 2001). The enigma of PV's role in fast-spiking neurons is rooted in its distinct kinetic properties. PV possesses a high affinity for Ca²⁺, with a dissociation constant (KD) in the low nanomolar range, approximately 10^−9^ M (Eggermann and Jonas, 2011, Schwaller, 2010). This high affinity means that once PV binds a Ca^2+^ ion, it holds on to it tightly. The paradox emerges from its slow binding kinetics. The intracellular concentration of Mg^2+^ is in the millimolar range, far exceeding the resting Ca^2+^ levels (Eggermann and Jonas, 2011, Schwaller, 2010). As Mg^2+^ and Ca^2+^ compete for the same binding sites on PV, a Mg^2+^ ion must first dissociate before a Ca^2+^ ion can bind. This competition is the rate-limiting step that dramatically slows down the apparent on-rate for Ca^2+^, making PV functionally akin to a slow calcium chelator like EGTA (Eggermann and Jonas, 2011, Schwaller, 2010).

### Functional consequence of slow kinetics

This "slow-onset" buffering has a unique effect on intracellular Ca^2+^ transients. During a rapid influx of Ca^2+^, such as that caused by an action potential, PV is too slow to bind a significant amount of Ca^2+^ during the initial rising phase. Consequently, it is an inefficient buffer of the peak amplitude of the transient (Chard et al., 1993). Instead, its primary action occurs after the peak has been reached. It acts as a high-capacity sink, binding Ca^2+^ and thereby accelerating the decay phase of the transient, effectively shortening the duration of the elevated Ca^2+^ signal (Lee et al., 2000, Schwaller, 2010). This kinetic signature is fundamentally different from that of a "fast" buffer, which would blunt the peak amplitude and slow the subsequent decay.

Why would fast-spiking interneurons express a slow buffer? One clue lies in the concentration and localization of PV. PV is predominantly located within the cytosol, where it is found in the soma, proximal dendrites, and axons, including presynaptic terminals (Celio, 1986, Lee et al., 2000). Strategic subcellular distribution positions it to influence a wide range of Ca^2+^-dependent processes, from the regulation of excitability at the soma to the modulation of neurotransmitter release at the synapse. When PV is present at very high concentrations, its buffering behavior changes. Pioneering work by Eggermann and Jonas directly measured PV levels in different neurons and found hippocampal PV interneurons contain PV on the order of ∼10–12 μM (on average), whereas cerebellar basket cells have about 50 × higher PV levels (Eggermann and Jonas, 2011, Schwaller, 2010). Therefore, PV’s effect on rapid synaptic events is relatively modest in hippocampal basket cells, but it significantly alters synaptic dynamics in cerebellar interneurons (Eggermann and Jonas, 2011). Modeling studies suggest that at high concentrations, PV can act almost like a fast buffer (comparable to BAPTA) by virtue of saturating local endogenous buffers and competing in the calcium nanodomains near release sites (Eggermann and Jonas, 2011). In other words, PV only exerts strong, immediate effects on Ca^2+^ transients when expressed at high levels (Eggermann and Jonas, 2011). At lower levels (like in most cortical/hippocampal PV neurons), PV might not bind the initial Ca^2+^ influx during an action potential but can still influence the residual Ca^2+^ that accumulates with repeated activity.

From a functional perspective, PV’s slow-on, high-capacity buffering may act as a lowpass filter for intracellular Ca^2+^ signals. It does not prevent the sharp rise of Ca^2+^ that triggers neurotransmitter release; instead, it sequesters Ca^2+^ on a longer timescale, shaping the decay phase of Ca^2+^ transients and the buildup of residual Ca^2+^ during high-frequency firing (Lee et al., 2000, Schwaller, 2010). By sequestering Ca²⁺ with a delay, PV shortens the duration of the post-spike calcium transient and limits how much residual Ca^2+^ carries over to influence subsequent spikes.

### Roles in synaptic plasticity and network dynamics

PV’s property in regulating calcium transient is crucial for modulating short-term synaptic plasticity, as demonstrated in a landmark study by Caillard et al. (Caillard et al., 2000). In PV knockout (PV^–/–^) mice, fast-spiking interneuron synapses showed a striking switch from paired-pulse depression to paired-pulse facilitation. Dialyzing a slow buffer (EGTA) into the presynaptic interneuron of a PV^–/–^ mouse could rescue the normal depression (Caillard et al., 2000), confirming that PV normally acts to curtail synaptic facilitation by buffering residual Ca^2+^. Consistent with this, PV-deficient hippocampal slices exhibit more robust synaptic responses during high-frequency stimulation. Vreugdenhil et al. found that in area CA1 of PV^–/–^ mice, trains of stimuli (>20 Hz) led to greatly increased facilitation of inhibitory postsynaptic currents (IPSCs) compared to wild-type, with IPSC amplitudes accumulating to over 200 % of initial response in the absence of PV (Vreugdenhil et al., 2003). In essence, removing PV “releases the brake” on repetitive GABA release, resulting in stronger inhibitory drive during network activity and paradoxically increased gamma oscillation power (Vreugdenhil et al., 2003). These findings resolved an apparent paradox in gamma rhythm research – while PV^+^ interneurons are required for generating gamma oscillations, the PV protein itself, by damping facilitation, limits the maximum gamma power. This suggests PV acts as a stabilizer, preventing runaway excitation-inhibition loops by high-pass filtering synaptic output.

Beyond presynaptic effects, PV’s calcium buffering might also affect postsynaptic integrative properties of interneurons. Fast-spiking PV^+^ interneurons often express calcium-sensitive potassium channels (e.g. SK and BK channels) that contribute to their afterhyperpolarization (AHP) and firing behavior (Bischop et al., 2012, Ma et al., 2023). By limiting the peak calcium reached during an action potential, PV could modulate the activation of these Ca^2+^-dependent K^+^ channels and thereby influence spike repolarization or frequency adaptation (Bischop et al., 2012). A computational model of striatal fast-spiking interneurons incorporating PV and SK channels illustrated that increasing PV concentration from 0 to 1.5 mM was predicted to slightly reduce the sustained firing rate of the neuron (e.g. steady-state firing frequency dropped from 39 Hz to 30 Hz as PV went from absent to very high) (Bischop et al., 2012). The mechanism is that PV buffers Ca^2+^ that would otherwise activate SK channels; without PV, more Ca^2+-^activation of SK leads to larger AHPs that slow the firing, whereas moderate PV prevents excessive SK activation, supporting high firing rates (Bischop et al., 2012). Interestingly, extremely high PV reduces the neuron’s firing frequency in the pattern of class 2 excitability (Izhikevich, 2006), pointing to an optimal range of PV for firing responsiveness. While direct experimental validation in hippocampal PV cells is challenging, these considerations underscore that PV can influence intrinsic excitability in addition to synaptic output – a point often overlooked when considering PV as “just” a marker.

### Activity-dependent regulation, gene expression, and neuroprotective roles

PV expression is not static; it is dynamically regulated by network activity, particularly during development and in response to experience. During normal postnatal development of the hippocampus, PV is not expressed at birth but begins to appear in interneurons toward the end of the second postnatal week (Doischer et al., 2008). A period that coincides with the maturation of inhibition and the emergence of network oscillations (Doischer et al., 2008, Su et al., 2017). L-type voltage-gated Ca^2+^ channels are highly expressed in PV interneurons and drive activity-dependent transcriptional programs that regulate PV expression (Jiang and Swann, 2005). Blocking L-type Ca^2+^ currents can delay or impede the maturation of PV^+^ interneuron (Jiang and Swann, 2005). In vitro and in vivo studies by Jiang and Swann showed that chronic blockade of L-type channels in developing hippocampus led to reduced PV levels and immature firing properties in interneurons (Jiang and Swann, 2005). Conversely, heightened network activity can promote PV expression (Caballero et al., 2020, Donato et al., 2013). This suggests a feedback loop that network activity stimulates Pvalb gene transcription, and the resulting increase in PV protein, which in turn tunes the network’s excitability and plasticity.

In the adult brain, recent experience can induce lasting changes in PV levels that affect learning and memory capacity (Chen et al., 2022, Donato et al., 2013). An elegant series of experiments by Donato and colleagues demonstrated a form of interneuron network plasticity, showing that PV basket cells can enter either a “low-PV” or “high-PV” configuration with functional consequences after certain experiences (Donato et al., 2013, Tripodi et al., 2018). Mice exposed to an enriched environment showed a sustained decrease in PV and GAD67 expression in CA1 basket cells (PV-low), whereas fear-conditioned mice showed an increase (PV-high) (Donato et al., 2013). These shifts were reversible and correlated with changes in the interneurons’ synaptic connectivity. Remarkably, the low-PV state was associated with enhanced structural synaptic plasticity and improved memory performance, while the high-PV state correlated with reduced plasticity and memory consolidation (Chen et al., 2022, Lu et al., 2014). Furthermore, during task learning (e.g., maze navigation), hippocampal PV networks initially shifted to a low-PV configuration, and then, after learning, reverted to a high-PV configuration (Chen et al., 2022), perhaps to stabilize the circuit and prevent further perturbation. This dynamic regulation indicates that PV is not merely a downstream marker of an interneuron’s identity, but also a lever that the brain can adjust to modulate the balance between plasticity and stability. Other studies also reported distinct functions of PV at low or high expression states, showing critical roles of the switch of PV’s states in the infralimbic (IL) mPFC in fear memory extinction and recall (Chen et al., 2022, Lu et al., 2014).

Another dimension of PV’s function is its role in metabolic support and neuroprotection. Fast-spiking PV neurons have high metabolic demands and experience large fluctuations in intracellular Ca^2+^ due to repetitive firing (Kole et al., 2022, Stoler et al., 2022). PV’s calcium-buffering capacity might protect these cells from Ca^2+^-mediated toxicity and manage the load on organelles like the mitochondria (Janickova et al., 2020). Calcium overload is a known trigger of cell injury and death (Dong et al., 2006), and PV (along with other Ca^2+^-binding proteins like calbindin) has been hypothesized to buffer excess Ca^2+^ and thereby confer neuroprotection (Mattson et al., 1991). Evidence for this in pathological conditions has shown that PV-rich neurons often resist acute excitotoxic challenges better than other neurons, presumably by limiting cytosolic free Ca^2+^ (Van Den Bosch et al., 2002). However, if the insult is too severe or prolonged, these interneurons can succumb, and often PV expression is one of the first things to change. For instance, after transient global ischemia, hippocampal PV interneurons initially survive the loss of their postsynaptic targets, but within days their PV levels drop and many cells degenerate. The loss of PV immunoreactivity in CA1 interneurons 5–14 days post-ischemia correlates with interneuron dysfunction and death (Himeda et al., 2005). Highlighting that PV might be a factor in their initial resilience and eventual vulnerability. A recent study found that PV-deficient interneurons show an age-accelerated increase in reactive oxygen species (ROS), linked to changes in mitochondrial distribution and Ca^2+^ handling (Janickova and Schwaller, 2020)*.* Interestingly, some ASD-like behavioral phenotypes manifest in PV^–/–^ mice already at one month of age (before emergence in ROS differences) (Janickova and Schwaller, 2020), indicating that PV loss causes circuit functional changes early on, while the oxidative stress builds more slowly. Nonetheless, the link between PV buffering and mitochondrial health is intriguing: PV may help PV neurons handle repetitive firing without engaging emergency Ca^2+^ clearance by mitochondria, thus preventing metabolic overload. In this way, PV can be seen as a protector of interneuron viability during intense activity, aligning with the idea that it shields neurons from Ca^2+^-induced toxicity. This neuroprotective aspect is especially relevant given that PV interneurons are notably vulnerable in certain neurological disorders (discussed later).

In summary, at the molecular level, PV serves as a slow but high-capacity Ca^2+^ buffer that can modulate synaptic release dynamics, intrinsic excitability, and activity-dependent gene expression. Its expression level is tuned by developmental and network activity, and it contributes to the resilience of fast-spiking interneurons under metabolic stress. The paradox of a slow buffer in fast-spiking neuron is resolved when one appreciates that PV’s role is not to blunt the initiation of fast signals, but to shape the integration and termination of those signals over time, thereby modulating synaptic plasticity, intrinsic excitability, and long-term cellular health.

## PV⁺ interneurons in the hippocampus: a diverse toolkit

The hippocampus contains several distinct subtypes of PV-expressing interneurons, each with specific morphology, connectivity, and functional roles. Despite their differences, they all share the fast-spiking phenotype, providing a powerful internal framework for testing hypotheses about PV's function (Bartholome et al., 2020, Yamada and Jinno, 2017) (Table 1).

**Table 1:** Summary of anatomical and functional characteristics of hippocampal PV^+^ interneuron subtypes.

Characteristic	Basket Cell (BC)	Bistratified Cell (BiC)	Chandelier Cell (ChC)	Oriens-Lacunosum Moleculare (OLM) Cell
Primary Location	stratum pyramidale (Lee et al., 2014) or proximal stratum oriens and radiatum (Booker and Vida, 2018)	stratum pyramidale (Klausberger et al., 2004)	stratum pyramidale or proximal stratum oriens and radiatum(Booker and Vida, 2018).	stratum oriens (Fernández-Arroyo et al., 2025)
Axonal Targeting	Soma, proximal dendrites of pyramidal cells (Lee et al., 2014)	Basal (in stratum oriens) and apical (in stratum radiatum) dendrites of pyramidal cells (Klausberger et al., 2004)	synapse exclusively with the axon initial segment (AIS) of excitatory pyramidal neurons	Distal apical dendrites (in stratum lacunosum-moleculare) of pyramidal cells (Fernández-Arroyo et al., 2025)
Key Molecular Markers	PV, Kv3.1/3.2, sometimes CCK (distinct population) (Lee et al., 2014, Southan and Robertson, 2000)	PV, sometimes SOM (Buhl et al., 1994)	PV (Cruz et al., 2003), cadherin 6 (Hardwick et al., 2005, Ishino et al., 2017) GABA(A) alpha(2) (Cruz et al., 2003) GAT1 (Cruz et al., 2003) PTHLH (Proddutur et al., 2023)	PV, Somatostatin (SOM), cholinergic receptor, nicotinic, 5HT3a (subset), Chrna2 (Mikulovic et al., 2015)
Primary Excitatory Input	Local pyramidal cells (feedback), CA3/EC afferents (feedforward) (Lee et al., 2014)	CA3 Schaffer collaterals (feedforward) (Klausberger et al., 2004)	Asymmetric inputs：a higher fraction in CA3 contralaterally but higher ipsilaterally in DG (Raudales et al., 2024)	CA3/EC inputs(Fernández-Arroyo et al., 2025; Leão et al., 2012)
Role in Oscillations	Gamma (γ) oscillation generation and synchrony (Lee et al., 2014)	Theta (θ) and Gamma (γ) modulation; Sharp wave–associated ripple episodes (Klausberger et al., 2004)	gamma and spindle oscillations coupling (Massi et al., 2012)	Theta (θ) rhythm generation and phase-locking (Sun et al., 2023)
Primary Circuit Function	Controls pyramidal cell spiking output; E/I balance (Lee et al., 2014)	Modulates integration of CA3 input via feedforward inhibition (Klausberger et al., 2004)	Not clear yet; Involved in feedback inhibition of granule cells in the dentate gyrus (Zitman et al., 2016)	Gates entorhinal cortex input; modulates dendritic integration; anxiety regulation (Mikulovic et al., 2015)

### Key subtypes and their circuit functions

• **Basket cell (BCs): the perisomatic gatekeepers**
  Basket cells are the most abundant and archetypal fast-spiking interneurons in hippocmapus. They are multipolar neurons located primarily in the pyramidal cell layer, extending a dense axonal arborization that forms "baskets" around the soma and proximal dendrites of principal cells (Johantges et al., 2025). This perisomatic targeting gives them powerful and precise control over the action potential generation of their targets (Halasy et al., 1996). Functionally, BCs are the primary drivers of network gamma oscillations (30–80 Hz) (Hadler et al., 2024). Through dense, reciprocal connections with pyramidal cells, they establish feedback and feedforward inhibitory loops that are essential for synchronizing neuronal ensembles and maintaining the local E/I balance (Dong et al., 2004).
• **Bistratified cells (BiCs): the dendritic sandwich**
  Bistratified cells provide a different form of dendritic inhibition. Their axons ramify in two distinct bands, targeting the basal dendrites in the stratum oriens and the apical dendrites in the stratum radiatum, thus "sandwiching" the pyramidal cell layer (Booker and Vida, 2018, Halasy et al., 1996). This axonal pattern is co-aligned with the glutamatergic Schaffer collateral inputs arriving from CA3 pyramidal cells. Consequently, BiCs are key players in the feedforward inhibitory circuit that modulates the strength and timing of this major intra-hippocampal pathway (Booker and Vida, 2018, Ferrante and Ascoli, 2015). While also being PV-positive and fast-spiking, they exhibit distinct electrophysiological properties from basket cells, such as a higher input resistance and a longer membrane time constant, suggesting a different computational role focused on input integration rather than output control (Booker and Vida, 2018)
• **Chandelier cells (ChCs)/ axo-axonic cells: the axon regulator**
  GABAergic chandelier cells (ChCs), also known as axo-axonic cells, were discovered in the 1970s, with the name given due to their resemblance to a chandelier (Gallo et al., 2020). The most distinctive morphological feature of chandelier cells (ChCs) lies in their highly specialized axonal morphology, with branches extending from the perikaryon form multiple vertically aligned "axonal cartridges," each composed of densely packed presynaptic boutons resembling the shades of a hanging chandelier (Somogyi et al., 1983). This structural specialization renders ChCs the only class of interneurons that specifically target the axon initial segment (AIS) of pyramidal neurons—the critical site for action potential initiation housing high-density voltage-gated sodium channels, functioning as the "decision center" for neuronal output signaling (Rasband, 2010). ChCs express PV and exhibit region-specific distribution patterns. They are most densely populated in layers 2/3 and 5 of the cerebral cortex, while being sparsely distributed near the pyramidal cell layers of the hippocampal CA1 and CA3 regions (Fairén and Valverde, 1980, Somogyi et al., 1983). Notably, hippocampal ChCs display shorter axonal cartridges and innervate twice as many pyramidal neurons compared to their cortical counterparts, a morphological adaptation that may align with the demand for local information integration within hippocampal circuits (Gallo et al., 2020).
• **Oriens-Lacunosum Moleculare (OLM) cells: the distal dendritic modulators**
  OLM cells have a distinct morphology and circuit position. Although canonically identified by the expression of somatostatin (SST), the OLM cell class is molecularly heterogeneous and contains a functionally distinct subpopulation that co-expresses PV (Fernández-Arroyo et al., 2025, Que et al., 2021). This distinction is critical, as SST-positive OLM cells are primarily associated with theta rhythms, whereas the PV-positive subset can fire in the gamma range, suggesting a different role in network coordination. OLM cell bodies reside in the outermost layer of the hippocampus (stratum oriens), and they project a long axon up to the innermost layer (stratum lacunosum-moleculare, SLM), the most distal dendritic layer of pyramidal cells (Leão et al., 2012). This is precisely where the main excitatory input from the entorhinal cortex arrives via the perforant path. By selectively inhibiting these distal dendrites, OLM cells act as gatekeepers, controlling the flow of cortical information into the hippocampus and modulating dendritic integration and plasticity (Leão et al., 2012). They are critically involved in the generation of and phase-locking to network theta oscillations (4–12 Hz) (Amilhon et al., 2015). Recent studies have also implicated ventral hippocampal OLM cells in the regulation of anxiety and risk-taking behavior, highlighting their role in emotional processing (Mikulovic et al., 2018). Meanwhile, studies regarding PV-expressing OLM subtypes in the hippocampus are still limited.

### Subtype connectivity

The connectivity of PV interneurons in hippocampal microcircuits is highly specialized. A single PV⁺ basket cell in CA1 can innervate dozens of pyramidal neurons, making multiple synapses onto each (Lee et al., 2014). These synapses are strategically clustered around the soma/axon, meaning a single basket cell can effectively phase-lock the firing of many nearby pyramidal cells simultaneously (Klausberger and Somogyi, 2008, Tukker et al., 2007)*.* PV basket and chandelier cells often fire in tight coordination with network oscillations, and they form interconnected networks among themselves as well (Massi et al., 2012). They are coupled by electrical synapses (gap junctions) on their dendrites, creating a fast interneuron network that can synchronize activity (Andrási et al., 2017, Traub et al., 2018). Early electron microscopy studies showed PV-positive interneurons in CA1 are extensively connected by gap junctions, which likely contributes to their ability to generate coherent high-frequency oscillations (Milicevic et al., 2024, Pernelle et al., 2018). PV interneurons also receive both excitatory and inhibitory inputs that are structured by layer. For instance, PV basket cells in CA1 get strong excitatory drive from local pyramidal cells and Schaffer collateral inputs, as well as inhibition from other interneurons including long-range septal GABAergic inputs (Bao et al., 2017, Lee et al., 2014). This positions PV cells as hubs that integrate local and extrinsic signals and rapidly distribute inhibitory outputs to orchestrate hippocampal ensemble activity.

A simplified wiring picture is that PV interneurons form a feed-forward and feedback inhibition system. In a feed-forward inhibition mode, PV cells activated by incoming entorhinal or CA3 inputs will inhibit pyramidal cells shortly-after the excitatory entorhinal input, tightening the time window for excitation (Willems et al., 2018). In the feedback inhibition mode, when pyramidal cells fire, local recurrent collaterals excite PV interneurons, which then promptly inhibit the upstream pyramidal cells (Kuramoto et al., 2022). By targeting soma/axons, basket and axo-axonic cells effectively clock the firing of pyramidal neurons, helping to generate rhythmic population oscillations such as the 30–100 Hz gamma oscillations (Kann et al., 2014, Tzilivaki et al., 2023). PV bistratified cells, while dendrite-targeting, also contribute to feed-forward inhibition during theta and gamma rhythms, ensuring that dendritic input integration is temporally sculpted (Klausberger et al., 2004).

### Physiological properties and functional roles

Physiologically, PV^+^ interneurons are defined by their fast-spiking phenotype. they can sustain high firing rates (>100 Hz) with very brief action potentials (Colgin et al., 2009). This is enabled by a specialized complement of ion channels, most prominently voltage-gated K^+^ channels of the Kv3 family, which allow rapid repolarization (Rosato-Siri et al., 2015), and Na^+^ channel subtypes like Nav1.1 that support high-frequency firing (Hu and Jonas, 2014). Indeed, Kv3.1/3.2 channels are highly enriched in PV interneurons and are critical for their fast AHPs and narrow spikes (Boddum et al., 2017, Chow et al., 1999). Genetic deletion of Nav1.1 (SCN1A) or certain Kv3 channels impairs PV cell excitability and is linked to epileptic phenotypes, reinforcing how essential these channels are for PV cell function (Catterall et al., 2010). PV interneurons also exhibit relatively low input resistance and fast membrane time constants (facilitating speedy voltage changes), and many have spike frequency adaptation mediated by Ca^2+^-activated SK channels (Maingret et al., 2008).

Synaptic terminals of the PV neurons are equipped with machinery for fast and precise GABA release, making them the "clockwork" of hippocampal networks (Kann et al., 2014). They are crucial for generating and maintaining oscillatory rhythms such as gamma (∼40 Hz) and contribute to sharp-wave ripple (∼140–200 Hz) events (Klausberger et al., 2003). During theta (4–8 Hz) oscillations, PV basket and axo-axonic cells in CA1 tend to fire at a particular phase coordinating with when pyramidal cells are least or most excitable (Klausberger et al., 2003). This coordinated firing enforces a temporal structure: PV interneurons provide a periodic inhibitory check that can synchronize the firing of pyramidal neurons into coherent oscillatory patterns. During active exploration, CA1 pyramidal cells often fire in gamma-time-scale bursts nested within theta cycles, and PV basket cells are instrumental in pacing those gamma cycles via rhythmic perisomatic inhibition (Bartos et al., 2002, O'Neill et al., 2006). Disruption of PV interneuron output typically leads to breakdown of gamma oscillations and deficits in precise spike timing of pyramidal cells. Conversely, optogenetic stimulation of PV cells at gamma frequency can impose or rescue gamma oscillations in the network (Sohal, 2012). In an Alzheimer’s model, driving PV cells at 40 Hz restored a deficient slow-gamma oscillation and improved memory performance (Etter et al., 2019). These observations underscore that PV interneurons are not only necessary but can be sufficient in some contexts to entrain network rhythms.

In terms of information processing, PV interneurons perform critical functions by controlling the temporal fidelity of neuronal firing and help sculpt receptive fields in space and time. Because basket cells can prevent or permit a pyramidal cell from firing based on the network oscillation phase, they effectively gate when that neuron can transmit information (Mysin et al., 2019). This gating is essential for processes like sequence encoding, phase precession, and coordinate transformations in the hippocampus (Gulyás et al., 2010). Additionally, by mediating lateral inhibition, PV cells can sharpen the spatial tuning of place cells and reduce noise (Valero et al., 2022). Some research suggests PV interneurons also contribute to pattern separation and pattern completion operations in the dentate gyrus and CA3 by limiting the spread of excitation and enforcing competition among principal cells (Leutgeb and Leutgeb, 2007, Morales et al., 2020, Tukker et al., 2013). The high-speed feedback inhibition from PV cells is especially important during memory recall and decision-making when timing is critical. In social memory retrieval, if the PV interneurons in ventral CA1 are inhibited, mice fail to distinguish familiar from novel conspecifics (Deng et al., 2019). This implies that PV interneuron activity during the retrieval phase is needed for proper read-out of stored memory, likely by coordinating hippocampal-neocortical interactions or controlling the timing of cell assemblies representing the memory (Xia et al., 2017).

Thus, PV interneurons are deeply embedded in the microcircuit motifs that underlie memory encoding, consolidation, and retrieval (Deng et al., 2019, Xia et al., 2017). They are not mere background “maintainers of inhibition” but dynamic participants that can modulate the flow of information depending on brain state.

### Development and plasticity

PV⁺ interneurons are generated in the embryonic medial ganglionic eminence (MGE) and migrate to the hippocampus during development (Yu et al., 2021). They undergo a protracted maturation: after reaching their positions, they gradually acquire PV expression and mature firing properties over the first 3–4 postnatal weeks (Ramsaran et al., 2023)*.* A key milestone is the onset of high-frequency oscillatory activity, which coincides with the functional emergence of PV interneurons (Bartholome et al., 2020, Yao et al., 2020). Disruption of normal activity patterns during this critical period, such as blocking NMDA receptors or L-type Ca^2+^ channels or by early life seizures, can lead to lasting alterations in PV cell development (Godoy et al., 2022, Guyon et al., 2021, Jiang and Swann, 2005). In rodents, by end of the first postnatal month, PV interneurons reach an adult-like state, characterized by strong perineuronal nets (PNNs) enwrapping their soma (Devienne et al., 2021). PNNs – specialized extracellular matrix structures – often form around PV cells and are thought to stabilize synaptic contacts and perhaps limit plasticity in adulthood (Fawcett et al., 2019). Notably, the formation of PNNs is an activity-dependent process that requires Ca²⁺ influx through L-type channels and AMPA receptors, creating a tight feedback loop where the maturation of PV^+^ neuron firing properties promotes the formation of the very structures that will stabilize its function (Dityatev et al., 2007). Functionally, PNNs are more than just structural stabilizers; they actively regulate the intrinsic excitability of the interneurons they surround and are critical for specific forms of memory. For example, the selective genetic removal of PNNs specifically from PV^+^ interneurons impair contextual fear memory, demonstrating a distinct role for this extracellular matrix in hippocampal-dependent learning (Alexander et al., 2025). The degradation of PNNs in disease states could therefore directly alters the calcium dynamics and excitability of PV^+^ neurons, contributing to network instability. Interestingly, interventions that enhance cortical plasticity through enzymatic removal of PNNs or environmental enrichment often coincide with reductions in PV levels, suggesting a link between the maturation state of PV cells (high PV, dense PNNs) and the closure of developmental critical periods (Mirzadeh et al., 2019, Pizzorusso et al., 2002).

Even in adult life, PV interneurons retain capacity for plastic changes as discussed with the Donato et al. findings (Donato et al., 2013). Synaptic plasticity can occur at inputs onto PV cells – for instance, excitatory synapses on PV interneurons can exhibit long-term potentiation or depression, though the rules often differ from typical Hebbian LTP/LTD in pyramidal cells (Udakis et al., 2020). One study found that in CA1 fast-spiking cells, the nonlinear integration of dendritic Ca^2+^ can determine the sign of plasticity: if a dendritic spike (Ca^2+^ plateau) occurs during pairing, the synapse undergoes LTD, whereas without it, LTP can occur (Udakis et al., 2020). This implies that PV interneurons can adjust the strength of their excitatory inputs based on firing patterns, which in turn modulates how effectively they are recruited during network events. Additionally, PV cells themselves can exhibit homeostatic plasticity (Zhao et al., 2024). If excitatory drive is chronically reduced, PV cells might downregulate PV expression or change their intrinsic properties to compensate (e.g. becoming more excitable). Conversely, excessive activity can lead to an increase in inhibition or even structural changes – for example, PV axon terminals can sprout or retract depending on network demand, and new inhibitory synapses can form or disappear on principal cells as needed (Micheva et al., 2021).

In summary, hippocampal PV interneurons form a diverse toolkit of inhibitory cells that enforce timing and gain control in the circuit. Their fast-spiking nature and powerful, precisely placed synapses enable them to synchronize neuronal ensembles and shape oscillations underlying memory processes. They undergo significant developmental maturation and can exhibit plasticity even in adulthood, with PV itself being a marker of their maturation state. These cells can also serve as barometers for circuit health – in disease states, their dysfunction or loss reverberates as network instability and cognitive dysfunction. Having painted a picture of PV interneurons’ identity and role in hippocampal circuits, we now return to our core question: how does the PV protein itself contribute to these neurons’ function, and what evidence distinguishes its role as a mere identifier versus an active regulator?

## Bridging PV protein to neuronal function

The co-expression of PV protein in fast-spiking interneurons is not a coincidence of nature. To address whether PV is more than an identity marker, researchers have employed a range of advanced techniques, which provide increasing evidence pointing towards a direct, causal role for the PV protein in shaping the functional signature of the neurons that express it (Table 2).

**Table 2:** Advanced methodologies for disentangling the PV protein-neuron relationship.

Genetic Tools		
Methodology	Specific Question Addressed	Key Insights & Representative Studies
*Conditional Knockout (cKO)*	Is PV functionally required during a specific developmental window or in a specific brain region (Yap et al., 2021)?	Allows separation of developmental vs. adult functions; demonstrates cell-type specific roles in behavior or disease model (Vogt et al., 2015).
*Point Mutation*	Is the Ca²⁺ buffering capacity of PV essential for its function, or is its mere presence sufficient (Tanner et al., 2005)?	To define the "active role" of protein according to its functional domains by decoupling the protein's presence from its primary biochemical function.
Functional Manipulation & Imaging		
Methodology	Specific Question Addressed	Key Insights & Representative Studies
*Optogenetics/Chemogenetics*	What is the causal role of PV^+^ neuron firing in generating network oscillations and driving behavior (Etter et al., 2019)?	Demonstrates that PV^+^ neuron activity generates gamma oscillations and influences cognitive behaviors like attention and memory consolidation (Bassant et al., 2005, Xia et al., 2017).
*2-Photon Ca²⁺ Imaging*	How does PV^+^ neuron activity acutely shape somatic and dendritic Ca²⁺ dynamics in target cells in vivo (Geiller et al., 2020)?	Enables direct visualization of how inhibitory inputs from PV^+^ cells sculpt the spatio-temporal patterns of network activity with single-cell resolution (Geiller et al., 2020).
Computational & Systems Biology		
Methodology	Specific Question Addressed	Key Insights & Representative Studies
*Single-Cell Multi-Omics (scRNA-seq) or Proteomics*	What is the full transcriptomic signature of a PV^+^ neuron, and how does it correlate with its morphological phenotype (Que et al., 2021)?	Identifies novel gene programs related to PV^+^ neuron function and dysfunction; reveals molecular diversity and identifies candidate markers for mature PV^+^ cells (Que et al., 2021).

### Advanced methodologies for disentangling the PV protein-neuron relationship

#### Precision genetic engineering

• **Conditional Knockouts (cKO):** The use of the Cre-loxP system, typically with a PV-Cre driver mouse line, allows for the deletion of the *Pvalb* gene or other genes of interest specifically in PV-expressing cells (Wang et al., 2022). By combining this with inducible Cre systems (e.g,Tet-On system), the target genes can be manipulated at specific developmental time points (Terzi et al., 2023). This is a major advance over global knockouts, as it can allow for the separation of PV's role during early development from its ongoing function in the mature circuit (Terzi et al., 2023).
• **Point Mutations:** The "gold standard" experiment for testing the active role hypothesis would involve engineering mice with point mutations in the *Pvalb* gene that selectively alter its function without deleting the protein (Tan et al., 2010, Tanner et al., 2005). Although still lacking, one could create a mutant PV with reduced affinity for Ca^2+^ or altered binding kinetics for Mg^2+^. This would definitively answer whether it is the mere presence of the protein (as a marker or structural element) or its specific Ca^2+^ buffering capacity that is critical for PV^+^ neuron function.

#### Functional manipulation & imaging

• **Optogenetics:** Modern tools allow more nuanced probing of PV interneuron function and, indirectly, PV protein’s role. Optogenetics has been pivotal: by using Cre-driver lines that express in PV cells (leveraging PV as a marker), researchers can selectively excite or inhibit PV interneurons during behavior (Etter et al., 2019, Park et al., 2020). While this doesn’t change PV protein levels, it tests the impact of activating those PV-expressing circuits. For instance, transiently silencing PV cells during the retrieval phase of a memory impairs recall, as shown in fear and social memory tasks (Deng et al., 2019, Xia et al., 2017). Stimulating PV cells at gamma frequencies can entrain local field potential oscillations and improve performance in cognitive tasks that depend on those rhythms (Etter et al., 2019). In one striking demonstration, acute optogenetic activation of hippocampal PV interneurons rescued impaired slow-gamma oscillation amplitude and improved memory in a contextual fear task (Etter et al., 2019). These experiments emphasize that the function conferred by PV interneurons is indispensable. However, they don’t directly tell us if PV protein is needed for that function – except that without PV, those interneurons might not be able to sustain such precise output.
• **Imaging:** Calcium imaging of PV interneurons, particularly with genetically encoded calcium indicators, has shed light on their in vivo activity patterns and calcium dynamics. Two-photon imaging in awake mice has revealed that PV cells can exhibit synchronous calcium spikes during network oscillations, and that their calcium transients are indeed brief – presumably in part due to PV’s buffering (Geiller et al., 2020). In one study of developing cerebellum, whole-cell recording and fluorescence imaging showed significantly faster Ca^2+^ decay kinetics for PV^+^ basket cells than stellate cells in early postnatal life. The maturation of these calcium dynamics coincided with the emergence of PV expression (Collin et al., 2005). In another study, imaging was used to compare Ca^2+^ transients in PV^+^ versus PV^–^ interneurons during high-frequency firing; PV^+^ cells had faster decay of calcium signals, consistent with PV’s effect (Lee et al., 2000, Vreugdenhil et al., 2003). While these sorts of experiments are challenging in hippocampus due to cell depths, they provide a more direct window into how PV shapes calcium handling during natural network activity.

#### Computational & systems biology

Single-cell transcriptomics and proteomics (omics) offer another approach: by profiling PV neurons at the molecular level, we can identify pathways and processes that depend on PV. Single-cell RNA sequencing of hippocampal interneurons consistently finds region-distinctive pattern of PV^+^ interneuron with different expression gene (DEGs) mainly related to RNA and protein metabolic pathway, and in some extent, neurodegenerative pathways at early-stage of 5XFAD mouse model, which is much different from SST^+^ interneuron and vglut^+^ excitatory neuron (Chen et al., 2025). Indeed, PV interneurons play a key role in Alzheimer disease. The change of PV interneuron’s proteomic may be another physiological marker of pathological impairment at early disease onset stage (Kumar et al., 2024). PV cells proteome show enriched expression of proteins related to mitochondrial, metabolic, synaptic, and neurodegenerative genetic risk and cognitive resilience, are all differentially regulated in human post-mortem brain proteomes or AD mouse models (Kumar et al., 2024). In addition, digging into the transcriptome dataset of PV neurons can also provide mechanistic insight for PV functioning (Moissidis et al., 2025). These studies are still emerging, but they lay the groundwork for “PV-omics”: understanding how the presence or absence of PV protein cascades into broader cellular changes and will potentially reveal molecular insights into the unique roles of PV in physiological and pathological states.

Experimental strategies are on the horizon to test PV’s role even more directly. For instance, an attractive idea is using inducible PV knockdown or overexpression in adult animals, to avoid developmental confounds (Wang et al., 2022). CRISPR-based tools might allow one to acutely suppress Pvalb expression in a targeted set of cells in an adult and examine physiological changes in real time (Mandegar et al., 2016, Meng et al., 2023). Conversely, inserting a floxed “stop” cassette in front of PV and then removing it with Cre at a chosen time could allow turning PV expression on after development (Belteki et al., 2005). These approaches could help answer whether PV is required developmentally for interneuron maturation, or can an interneuron develop normally without PV and show deficits later? So far, conventional PV^–/–^ mice suggest mostly postnatal functional deficits rather than gross developmental errors, but more precision is needed.

Another strategy is pharmacological – can we mimic or replace PV’s buffering? This is tricky, as no drug will perfectly act like PV, but certain calcium chelators can be loaded into cells (Tymianski et al., 1993). Alternatively, one could try to potentiate the downstream effects of PV loss. For example, if PV loss causes more residual Ca^2+^ and excessive transmitter release, could a partial block of presynaptic Ca^2+^ channels normalize transmission? Some antiepileptic drugs (like levetiracetam) reduce synaptic release probability (Meehan et al., 2011), and might thereby counteract the facilitation seen with PV loss. These concepts are speculative but represent ways to tie mechanism to potential therapy.

### Probing PV necessity: insights from knockout and transgenic studies

The PV knockout mouse provides a clear test of PV’s necessity for various cellular and network properties. As reviewed earlier, PV knockouts have essentially normal development and positioning of interneurons, with PV neurons still firing fast spikes, but subtler changes abound (Albéri et al., 2013, Csillik et al., 2010). PV^–/–^ mice show increased facilitation at interneuron synapses and heightened gamma oscillation power in acute slices (Schwaller et al., 2004, Vreugdenhil et al., 2003). Behaviorally, PV^–/–^ mice are viable and overtly normal in many basic research. One study reported that PV knockout mice display abnormalities encompassing all core domains of autism (social interaction deficits, repetitive behaviors, and communication impairments), alongside changes in neural oscillations and synaptic plasticity (Wöhr et al., 2015). Another line of research noted that knockdown of PV in the anterior cingulate cortex caused increased anxiety-like behavior and enhanced fear learning, possibly due to altered inhibitory neural circuits (Jahangir et al., 2025). These findings imply that PV, while not required for interneurons to exist or fire, does influence higher-order network function and behavior, supporting an active role. It might be hypothesized that PV knockout can trigger homeostatic compensatory mechanism, such as upregulation of other Ca^2+^ buffers like calbindin, or changes in synaptic density, so not every effect can be cleanly ascribed to the acute loss of PV’s buffering. However, it remains a risk factor in many diseases, where specific phenotypes in PV^–/–^ mice mirror aspects of human disorders (like increased baseline gamma power in ASD models (van Diessen et al., 2015; Wöhr et al., 2015)), which strengthens the case that PV levels modulate circuit dynamics in meaningful ways.

### Establishing causality: acute rescue and dynamic manipulation experiments

One powerful piece of evidence for PV’s causal role is the acute rescue experiments done ex vivo. Eggermann and Jonas showed that if you take a PV^–/–^ slice and load a presynaptic neuron with recombinant PV you can restore normal short-term plasticity (Eggermann and Jonas, 2011). The facilitated IPSCs in PV^–/–^ terminals convert back to wild-type depression once PV protein is introduced, confirming that it was the absence of PV rather than irreversible developmental change that caused synaptic dynamics. Conversely, acutely chelating Ca^2+^ in wild-type PV neurons with exogenous buffer mimics the effect of PV (Eggermann and Jonas, 2011). These kinds of manipulations, though technically challenging, demonstrate a direct functional linkage between PV protein and the cell’s electrophysiological behavior. In line with this, viral overexpression of PV in neurons that normally do not express this protein has been attempted by some groups while results are mixed (exogenous PV expression is sometimes difficult to achieve in adult neurons), such experiments generally find that introducing PV can dampen GABA/glutamate expression ratio in the targeted cells (Zeng et al., 2023, Zeng et al., 2024), while the effects on short-term facilitation and firing properties awaits further study.

### The PV system in disease: a hub of vulnerability

The very properties that make PV^+^ neurons powerful regulators of circuit function also render them exceptionally vulnerable to pathological insults. Dysfunction of the PV system is a remarkably convergent finding across a wide range of brain disorders that affect hippocampal and cortical circuits (Marín, 2012) (Table 3).

**Table 3:** Summary of PV system dysregulation in major neurological and psychiatric disorders.

Disorder	Key Brain Region(s)	Observed PV^+^ Neuron Pathology	Observed PV Protein Pathology	Functional Consequence	Evidence for Causality
Schizophrenia	Prefrontal Cortex, Hippocampus (Enwright et al., 2016, Shih et al., 2025)	Reduced GAD67; altered connectivity; PNN deficits (Enwright et al., 2016, Fung et al., 2010)	Reduced mRNA and protein expression (Konradi et al., 2011, Ruden et al., 2021)	Impaired gamma oscillations; cognitive deficits (e.g., working memory) (Gonzalez-Burgos et al., 2015)	Animal models mimicking PV deficits replicate symptoms; some genetic risk factors linked to PV cell development (Mukherjee et al., 2019, Santos-Silva et al., 2024).
Epilepsy	Hippocampus, Amygdala (Sun et al., 2024)	Selective cell loss, particularly in the hilus and CA1/CA3 (Mátyás et al., 2021)	Reduced expression in surviving neurons (Scotti et al., 1997)	Network hyperexcitability reduced seizure threshold; generation of seizures (Scotti et al., 1997)	PV protein is neuroprotective; chemogenetic or optogenetic manipulation of PV^+^ cells can modulate seizures. (Assaf and Schiller, 2016, Chen et al., 2020)
Alzheimer's Disease	Hippocampus, Cortex (Smeralda et al., 2024)	High vulnerability and early degeneration(Mahar et al., 2016; Ruden et al., 2021)	Downregulation associated with pathology (Kumar et al., 2024)	Network hyperactivity; impaired gamma/theta oscillations; memory deficits (Ahnaou et al., 2017, Dickerson et al., 2005)	Restoring PV^+^ neuron activity can prevent memory loss in AD models; PV^+^ hyperexcitability may prime for Aβ toxicity (Hijazi et al., 2020a).
Autism Spectrum Disorders (ASD)	Cortex, Hippocampus (Contractor et al., 2021)	Reduced cell numbers in some models; altered development(Ariza et al., 2018; Hashemi et al., 2017)	Reduced expression in some models (Filice et al., 2016, Wöhr et al., 2015)	E/I imbalance; social and cognitive deficits; repetitive behaviors (Hashemi et al., 2017)	Global PV knockout mice show ASD-like behaviors; cKO of risk genes (e.g., *Pten*) in PV^+^ cells is sufficient to cause symptoms (Kwon et al., 2006, Shin et al., 2021).

#### A common pathological hub

• **Schizophrenia:** A leading hypothesis in schizophrenia research posits that the disorder arises from a deficit in prefrontal E/I balance, with a specific impairment of PV^+^ interneurons (Chamberlin et al., 2023). Post-mortem studies consistently show reduced expression of PV and the GABA-synthesizing enzyme GAD67 in the prefrontal cortex and hippocampus (Fung et al., 2010, Santos-Silva et al., 2024). Mechanistically, this reduction in GABA synthesis weakens the perisomatic inhibition that PV^+^ neurons provide, impairing their ability to precisely synchronize pyramidal cell firing and thereby directly causing the attenuated gamma-band oscillations seen during cognitive tasks (Gonzalez-Burgos et al., 2015). Furthermore, a selective reduction in hippocampal PV expression has been shown to be sufficient to induce ventral hippocampal hyperactivity and downstream dopamine system hyperfunction, linking the PV deficit directly to the positive symptoms of schizophrenia (Boley et al., 2014). This cellular deficit is thought to underlie the impairments in gamma oscillations observed in patients, which in turn contribute to the profound cognitive deficits, such as working memory impairment, that are core features of the illness (Gonzalez-Burgos et al., 2015).
• **Epilepsy:** In temporal lobe epilepsy, the most common form of focal epilepsy in adults, there is often a selective loss of PV^+^ interneurons in the hippocampus (Lee et al., 2024). This loss of powerful perisomatic inhibition is believed to be a key factor contributing to the network hyperexcitability that generates seizures (Lee et al., 2024). This hyperexcitability can arise directly from impaired PV^+^ interneuron biophysics; for example, loss-of-function mutations in the NaV1.1 sodium channel, which is predominantly expressed at the axon initial segment of PV^+^ interneurons, compromises their ability to sustain high-frequency firing, leading to a failure of inhibitory control and a lowered seizure threshold (Ogiwara et al., 2007). Conversely, the PV protein itself appears to be neuroprotective; its presence helps shield neurons from the excitotoxic Ca^2+^ overload that occurs during seizures, suggesting that its downregulation or loss could exacerbate pathology (Niquet et al., 2005, Van Den Bosch et al., 2002).
• **Alzheimer's Disease (AD):** PV^+^ neurons appear to be one of the earliest and most vulnerable cell types affected in AD (Hijazi et al., 2023). Their dysfunction contributes to the network hyperactivity, aberrant gamma oscillations, and memory impairments seen in both AD patients and mouse models (Jiménez-Balado and Eich, 2021). Some evidence suggests that an initial state of PV^+^ neuron hyperexcitability may render the hippocampus more vulnerable to subsequent amyloid-beta toxicity, potentially acting as a priming factor for the disease (Hijazi et al., 2020b), while restoring PV interneuron activity shows beneﬁcial effects on memory and hippocampal network activity, and even reduces amyloid plaque deposition (Hijazi et al., 2020a)
• **Autism Spectrum Disorders (ASD):** Evidence from both human post-mortem tissue and animal models points to a reduction in the number or function of PV^+^ interneurons in ASD (Ariza et al., 2018, Hashemi et al., 2017). Recent findings provide direct molecular pathways for this dysfunction, showing that certain ASD-associated mutations can lead to hypomyelination specifically of PV^+^ interneurons, which reduces their excitability and disrupts the gamma oscillations necessary for sensory processing (He et al., 2025). Other pathways, such as astrocyte-neuron signaling via the EphB2/ephrin-B1 complex, are also implicated in regulating the formation of PV^+^ inhibitory synapses, the disruption of which leads to ASD-like phenotypes (Sutley-Koury et al., 2024). The causal link is further supported by findings that PV^-/-^ mice exhibit ASD-like behavioral deficits (Janickova et al., 2020). Furthermore, conditional knockout of prominent ASD risk genes, such as *Pten*, specifically within PV^+^ neurons is sufficient to recapitulate core behavioral phenotypes of autism in mice, including social deficits and repetitive behaviors (Shin et al., 2021).

### Correlation vs. causation: a critical question

A central issue in interpreting the consistent involvement of PV^+^ interneurons in brain disorders is whether their dysfunction is a primary cause of the disease or a secondary consequence of a broader pathological process. While PV^+^ neuron loss could simply be a downstream effect of a hostile cellular environment (due to excitotoxicity or oxidative stress), a growing body of evidence points towards a more direct, causal role.

Two key lines of reasoning bolster the argument for causality. First, the intrinsic vulnerability of PV^+^ neurons positions them as a potential early point of failure (Ruden et al., 2021). Their high metabolic demands, extensive connectivity, and constant high-frequency activity make them exquisitely sensitive to a wide range of insults (Kann et al., 2014, Ruden et al., 2021, Yao et al., 2020). Therefore, PV downregulation or cell loss could be an early event in a disease cascade that subsequently triggers broader network dysfunction.

Second, and more convincingly, rescue experiments are accumulating to provide direct causal evidence. As highlighted across multiple disease models, techniques like optogenetics and chemogenetics that restore the activity of dysfunctional PV^+^ neurons can reverse cognitive and behavioral deficits. This demonstrates that restoring function to this specific cell type can have therapeutic benefits, strongly supporting a causal role for their dysfunction in the disease state.

Considering all the evidence, a consensus is emerging that PV is both a phenotypic marker and a functional regulator. It is a marker in the sense that it demarcates a subclass of interneurons with a specific developmental origin, morphology, and physiology. Many studies use PV immunostaining or PV-Cre lines to identify or manipulate these cells, effectively treating PV as an inner label (Topolnik and Tamboli, 2022). However, as reviewed, altering PV levels changes the behavior of these neurons in nontrivial ways. The apparent paradox of a slow buffer in a fast neuron resolves when one appreciates that PV provides temporal tuning – it endows fast interneurons with the ability to sustain rapid firing without saturating, to recover quickly between bursts, and to limit excessive facilitation that could destabilize network rhythms. In doing so, PV helps define the interneuron’s functional signature

From a broader perspective, PV exemplifies how a molecular identity gene can serve a dual purpose. It marks membership in a cell type, but also actively shapes the role of this cell type in the circuit. This duality prompts deeper questions. Did interneurons evolve PV expression because it conferred a fitness advantage for certain neural computations (e.g. high-frequency oscillations) (Hadler et al., 2024)? Could other cell types benefit from expressing PV, or conversely, do PV cells in some conditions reduce PV to adapt to new demands (as in the Donato low-PV network for enhanced plasticity) (Donato et al., 2013)? The answers are being unraveled gradually. What is clear is that the presence of PV protein influences interneuron function in way that is context-dependent.

## Conclusion

**Fig. 1:**
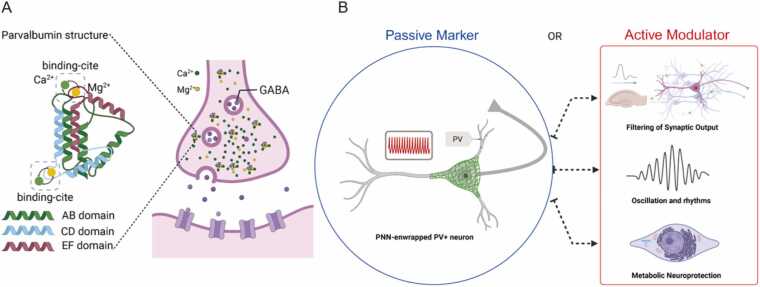
Parvalbumin: from molecular identity to active modulator in fast-spiking interneurons. (A) Molecular structure of parvalbumin, featuring AB, CD, and EF domains. The CD and EF domains contain EF-hand motifs that competitively bind Mg²⁺ at rest and Ca²⁺ during neuronal activity. (B) Functional roles of parvalbumin extend beyond its use as a cellular marker. It actively modulates synaptic output, fine-tunes network oscillations, and provides metabolic neuroprotection, thereby sustaining the high-frequency firing capabilities of PV⁺ interneurons.

Parvalbumin in hippocampal interneurons serves as both a signature of cell identity and a crucial factor in neuronal function. Far from being a passive marker, PV plays an active role in filtering synaptic output, regulating oscillation and rhythms, and providing metabolic neuroprotection (Fig. 1). This duality can be framed as a “molecular gearshift”: PV^+^ interneurons can shift between high plasticity, low-PV states and high-fidelity, high-PV states depending on experience and network needs, thereby balancing flexibility and stability in neural circuits (Chen et al., 2022). The slow calcium buffer paradox is resolved in this light – PV’s slow binding is precisely tuned to the fast-spiking interneuron’s requirements, allowing immediate signals to pass while integrating their effects over longer timescales to prevent synaptic and cellular overload. In hippocampal networks, this means PV interneurons can reliably pace rhythms like gamma and theta, constrain aberrant excitation, and yet also undergo configuration changes to facilitate learning when needed (Hadler et al., 2024).

Clinically, the recognition of PV’s functional importance has several implications. For one, PV interneurons (and by extension PV protein) represent a promising target for intervention in disorders characterized by E/I imbalance and oscillation dysfunction (Selimbeyoglu et al., 2017). Enhancing PV interneuron function that protect the system from oxidative stress, or leveraging behavioral therapies that modulate PV network states could be powerful therapeutic strategies.

Outstanding questions remain. A critical future direction is to examine PV's role within the broader context of the entire interneuron ensemble, where it acts in concert with other inhibitory cell types like those expressing somatostatin or VIP (Tzilivaki et al., 2023). Furthermore, it is important to consider how PV's function compares to that of other CaBPs, such as calretinin, which mark interneurons with different physiological profiles (Saffari et al., 2019, Schwaller, 2007).

In summary, the parvalbumin paradox exemplifies how evolution repurposes molecular tools for specific circuit functions. PV both identifies a cell type and equips it with a distinctive biophysical property: the capacity for rapid, repeated action without self-destruction or desynchronization. Hippocampal PV interneurons leverage this to perform their role as precision timers in the neural orchestra. Ongoing research, armed with modern techniques, is poised to deepen our understanding of PV, potentially revealing general principles of how molecular phenotype influences neural computation. By appreciating molecules like PV as more than mere labels, we unlock new strategies to tune brain rhythms and cognitive function in health and disease.

## CRediT authorship contribution statement

**Na Zhang:** Writing – review & editing, Writing – original draft, Investigation. **Huiqian Huang:** Writing – review & editing, Writing – original draft, Supervision, Project administration, Funding acquisition, Conceptualization. **Xiao-Ming Li:** Writing – review & editing, Conceptualization. **Bo-Wu Hu:** Writing – review & editing.

## Declaration of Competing Interest

The authors declare that they have no known competing financial interests or personal relationships that could have appeared to influence the work reported in this paper.
